# Mood after Moderate and Severe Traumatic Brain Injury: A Prospective Cohort Study

**DOI:** 10.1371/journal.pone.0087414

**Published:** 2014-02-04

**Authors:** Linda Valk-Kleibeuker, Majanka H. Heijenbrok-Kal, Gerard M. Ribbers

**Affiliations:** Department of Rehabilitation Medicine and Physical Therapy, Erasmus MC, University Medical Center Rotterdam, and Rijndam Rehabilitation Center, Rotterdam, The Netherlands; University of Pittsburgh, United States of America

## Abstract

**Objective:**

To evaluate the course of mood and identify its determinants up to 3 years after moderate to severe traumatic brain injury (TBI).

**Design:**

Prospective cohort study.

**Patients:**

Patients hospitalised with moderate to severe TBI, who survived until hospital discharge.

**Methods:**

At 3, 6, 12, 18, 24, and 36 months post-injury, mood was assessed with the Wimbledon Self-Report Scale (WSRS) in the home environment. Motor and cognitive outcome were assessed with the Functional Independence Measure (FIM), and the Functional Assessment Measure (FAM), respectively. Repeated measurements analysis was performed to determine the course of mood over time and its determinants.

**Results:**

A total of 98 patients (72% men), aged 33 (SD 12.9) years, 78% with severe TBI, was included. Mood did not change until 18 months post TBI, after which it significantly improved (p = 0.016). The FIM score significantly improved up to 18 months post-TBI (p = 0.012) and the FAM score up to 12 months post-TBI (p = 0.000), after which both remained stable. In univariable analyses, time post TBI (*β* = −0.04, p = 0.008), initial discharge destination (*β* = 2.13, p = 0.010), FIM (*β* = −0.22, p<0.001) and FAM (*β* = −0.29, p<0.001) were significant predictors of mood. In a multivariable mixed model, time post TBI, FAM score, and discharge destination were the strongest predictors of mood. Higher FAM scores were related to better mood scores (*β* = −0.28, p<0.001). Patients initially discharged home tended to have better mood scores over time than patients first treated in inpatient rehabilitation centers or nursing homes (*β* = 1.27; p = 0.071).

**Conclusion:**

Mood starts to improve 18 months after TBI when motor and cognitive outcome have stabilized. Time post TBI, cognitive outcome and initial discharge destination are the strongest predictors of mood up to 3 years after TBI. These data suggest that mood scores of patients with moderate and severe TBI should be frequently monitored, especially in rehabilitation centers and nursing homes.

## Introduction

In Europe, about 1.6 million traumatic brain injury (TBI) patients are admitted to hospital on a yearly basis. While national incidence rates may vary, the incidence rate of TBI for the European population is 235/100,000 with 66,000 deaths per year [1], [2]. The outcome after TBI can vary from complete recovery to death, with many patients having long-term disabilities. Especially after severe TBI disabling cognitive, behavioural, emotional and sensorimotor impairments can occur [3].

Psychiatric disorders often complicate recovery and rehabilitation from TBI, and the most frequently diagnosed psychiatric disorder after TBI is depression [4]. Depression may hinder the achievement of optimal rehabilitation goals and the successful reintegration of the patient into the home, family, community, and work environment [5].

Data on the occurrence of depression after TBI are not consistent. A prospective study, reported depression rates in adults with mild to severe TBI of 53% in the first year after hospital discharge [6]. Another study in an inpatient rehabilitation cohort examined major and minor depression in relation to post-injury societal participation: 22% reported minor depression and 26% reported major depression at 1 year post-TBI. Both major and minor depression were associated with sex (women), age (younger), pre-injury mental health treatment, substance abuse and cause of injury (intentional) [7]. Six months post-injury emotional state, and cognitive and everyday functioning, are reported to be interrelated [8]. Psychosocial stressors, maladaptive coping styles, and lack of work/fear of job loss are the most important predictors for the development of a depression after TBI [9]. One retrospective study reported that at (on average) 14.1 years post onset, 45.3% of the severe TBI patients were depressed [10]. In a long-term prospective study examining mild to severe TBI, a depression rate of 17% was found 3–5 years post onset. Educational level, pre-injury unstable work history, and alcohol abuse predicted depression [11].

Depression is an important problem and vulnerability to develop depression after TBI may be determined by a complex interplay between genetic, developmental, psychosocial and TBI- related characteristics [12]. Long-term prospective follow-up studies on depression after TBI are scarce. Most studies have focused on short-term outcomes with a maximum follow-up of 6–12 months post-injury, and have a retrospective design.

The current study focuses on the long-term time course of mood after moderate and severe TBI. It is part of the Rotterdam TBI study, a prospective study with a follow-up of 3 years post-onset. The first aim is to prospectively follow the course of mood scores in relation to physical and cognitive functioning up to 3 years after moderate and severe TBI, and to investigate the predictors of these mood scores.

In the Netherlands, TBI patients are discharged from the hospital to their home with or without outpatient rehabilitation [1], to an inpatient rehabilitation center [2], or to a nursing home [3]. Triage depends on the intensity of the rehabilitation program the patient can tolerate, the expected recovery rate, and the social context of the individual patient. The second aim is to explore if there is a difference in the course of mood over time between these groups up to 3 years after TBI, taking into account physical and cognitive functioning. No study has investigated the relationship between initial discharge destination from the hospital and mood scores over time. Also, most studies focus on inpatient rehabilitation cohorts, whereas the present study includes both inpatient and outpatient groups. Our expectation is that TBI patients initially discharged to an inpatient rehabilitation center or nursing home will have worse mood scores than patients directly discharged to their homes.

## Methods

### Ethics Statement

All eligible TBI patients and their family members or legal representatives received verbal and written information about the study. If possible, patients were asked to give written informed consent. If patients were unable to sign, a legal representative provided written informed consent and the patients were asked to sign the informed consent form at a later time. The Medical Ethics committee of Erasmus MC approved this study (MEC 174.273/1998/183).

### Study Population

In this study, patients with moderate or severe TBI (Glasgow Coma Scale; GCS 3–12) were included between January 1999 and April 2004 at three Dutch acute care hospitals, all large trauma centers: Erasmus MC, Rotterdam (January 1999 to April 2004; Medical Center Haaglanden, the Hague (January 2003 to February 2004); and University Medical Center Utrecht, Utrecht (April 2003 to February 2004) [13], [14]. Patients were discharged from the acute care hospital to their homes (with or without outpatient rehabilitation), to an inpatient rehabilitation center or to a nursing home.

Inclusion criteria were: (*i*) admission to a hospital for moderate (GCS 9–12) or severe (GCS 3–8) [15] TBI; (*ii*) age 16–67 years; and (*iii*) survival until discharge from the hospital. Exclusion criteria were: (*i*) insufficient knowledge of the Dutch language to participate in the study; or (*ii*) serious pre-traumatic neurological, oncological, or systemic disorders (e.g. spinal cord injury, cancer or psychiatric disorder) that may interfere with TBI-related disability assessments.

### Procedure

Patients were followed prospectively at 3, 6, 12, 18, 24, and 36 months post-injury. Two certified research psychologists collected data by means of structured interviews at the patients’ homes, at the rehabilitation center or at the nursing home where the patient stayed at that time. If it was not possible to interview the patient, a family member or professional caregiver was interviewed. This was not applicable for the assessment of mood for which a self-report questionnaire was used. Self-report scales were only completed by patients themselves.

### Measurement Instruments

#### Baseline measurements

Baseline characteristics were collected during the hospital stay by the hospital staff. Socio-demographic characteristics included age, gender, marital status, pre-injury work status (employed versus not employed) and pre-injury educational level.

TBI severity was based on the lowest GCS score, measured within the first 24 h of hospital admission. Length of stay (LOS) in hospital, rehabilitation center, and/or nursing home were registered. We classified the discharge destinations after hospital discharge in three categories: home (the **Home** group), an inpatient rehabilitation center or a nursing home (together these latter two form the **Rehab** group).

#### Emotional status

Mood was assessed using the Wimbledon Self-Report Scale (WSRS), which is especially suitable for neurological patients [16]. This self-rating scale addresses only the patient’s feelings and avoids enquiry about somatic symptoms, memory and concentration problems. WSRS scores appear to be unaffected by sex or age within the 18–80 year range [16]. The rates of false positive (about 4%) and false negative (about 6%) outcomes are acceptably low [16]. The survey consists of 30 adjectives and phrases describing feelings. Of these, 24 describe unpleasant feelings (e.g. ‘miserable’, ‘as if I am being punished for something’) and 6 describe pleasant feelings (e.g. ‘happy’, ‘content’). Total scores range from 0–30, with high scores indicating pervasive unpleasant feelings. Scores of 0–7 are considered as normal, scores of 8–10 as borderline and scores of at least 11 represent a clinically significant mood disturbance [16]. We used a validated Dutch translation of the WSRS questionnaire [17]. The intra-class correlation coefficient and the squared weighted kappa of this instrument were both 0.75 in the TBI population of this study [13]. WSRS scores were collected at each measurement time if patients were no longer staying in hospital, rehabilitation center or nursing home. Thus, mood was assessed only after final discharge to the home environment, so that institutional factors could not influence mood scores.

### Physical and Cognitive Functioning

Post-acute functional measures included the Functional Independence Measure and Functional Assessment Measure (FIM and FAM), measured at time of discharge from the acute care hospital and during follow-up. The FIM and FAM, which has good reliability and validity [18]–[21] consists of 30 items that are evaluated on a 7-point scale (completely independent to totally dependent). The 18 items of the FIM evaluate especially motor functions such as self-care, sphincter control, transfers, and locomotion, whereas the 12 items of the FAM primarily evaluate cognitive functions, such as communication, psychosocial adjustment, orientation and attention. The FIM motor scale ranges from 18 (totally dependent) to 126 (totally independent). The FAM cognitive scale ranges from 12 (totally dependent) to 84 (totally independent). FIM and FAM scores were rated by certified research psychologists at each measurement time.

### Statistical Analyses

Descriptive analyses were performed for the total group; means and standard deviations (SD) were calculated for continuous variables, and the total number and percentages for categorical variables. Means (SD) of age, GCS score, and LOS were also calculated for subgroups of patients with complete data versus patients with missing data on the WSRS or FIM at 3 and 36 months to study potential selection bias.

The course of mood and functional outcomes over time were calculated using linear mixed models with repeated measurements. These analyses take into account that multiple measurements within subjects are correlated. This procedure is very flexible in handling missing values, by estimating the covariance parameters in the data.

Using univariable and multivariable linear mixed models we evaluated the effect of potential predictors, measured at baseline and during follow-up, on the mood scores over time. Potential predictors of mood included time of measurement, patient characteristics (age, gender, living with partner, educational level, pre-injury employment status), injury severity variables (LOS, TBI severity: moderate (GCS 9–12) versus severe (GCS 3–8)), discharge destination (Home versus Rehab), and time varying functional outcomes (FIM and FAM), measured at the same times as the mood scores. All variables that were significant with a p-value <0.05 in the univariable models were selected for the multivariable model. Using backward variable selection, variables were removed from the multivariable model if the p-value >0.10. Model fit was checked using the likelihood ratio test and Aikaike’s information criterion. Lower model fit statistics indicate a better fit. All analyses were performed using SPSS version 18.

## Results

### Study Population

In total, 549 patients with TBI were screened in the hospital. Of these, 153 patients died during hospitalization and 229 patients were excluded: 90 patients did not fit the age range, 46 patients had mild TBI, 45 patients had severe co-morbidity, 42 patients were transferred to another area, and 6 patients did not have a sufficient command of the Dutch language. Therefore, 167 eligible patients were available, of which 113 consented to participate. Of these participants, 98 completed the WSRS questionnaire after final discharge to their homes and were included in the analyses.

The mean age of the study population was 33 (SD 12.9) years, the majority were men (72%) and 78% had severe TBI. Patient characteristics are presented in Table 1.

**Table 1:** Characteristics of patients with moderate to severe traumatic brain injury (N = 98).

Patient characteristics	Mean (SD)	N (%)
Age, years	33 (12.9)	
Gender, men		71 (72)
Married/living with partner pre-injury*		47 (48)
Employed pre-injury*		80 (83)
Educational level, low*		46 (47)
TBI Severity		
*Moderate (GCS 9–12)*		22 (22)
*Severe (GCS 3–8)*		76 (78)
Hospital discharge to:		
*Home*		45 (46)
*Rehabilitation center*		41 (42)
*Nursing home*		12 (12)
Length of stay, days		
*Hospital*	39 (28.6)	
*Rehabilitation center*	93 (21.7)	
*Nursing home*	154 (30.8)	
FIM at hospital discharge*	102 (24.6)	
FAM at hospital discharge*	62 (15.2)	

*Data missing for: pre-injury work status (*n* = 15), living status (*n* = 13), educational level (*n* = 14), FIM (n = 18) and FAM (*n* = 19).

SD: standard deviation; TBI: traumatic brain injury; GCS: Glasgow Coma Scale; FIM: Functional Independence Measure; FAM: Functional Assessment Measure.

### Course of Mood Score, FIM and FAM Over Time

**Figure 1:**
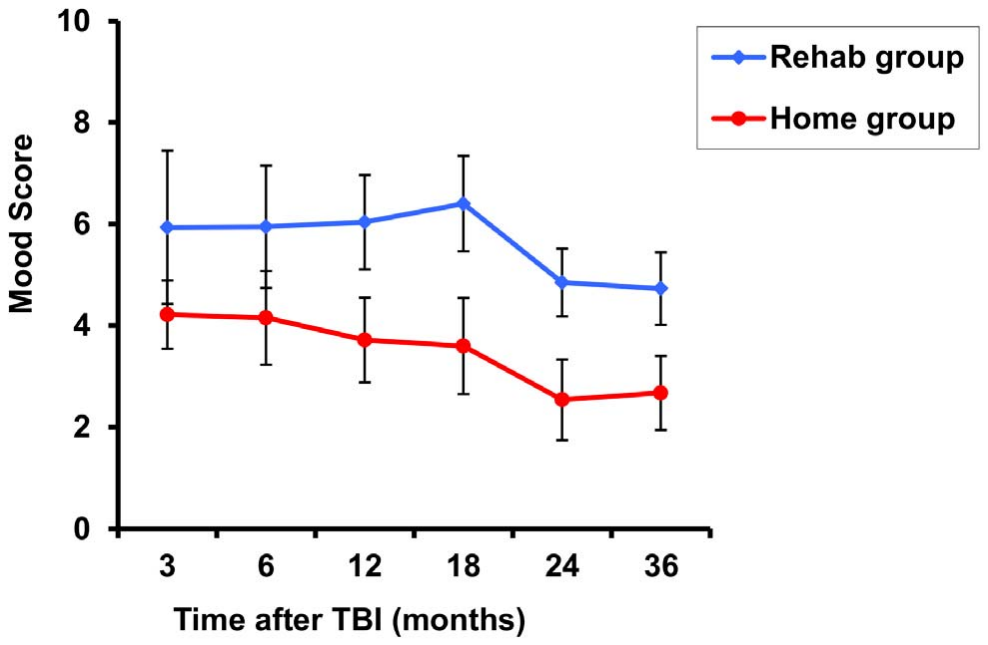
Estimated mean (±1 SD) Wimbledon Self-Report Scale (WSRS) scores over time in the Rehab group (blue line; n = 53) versus the Home group (red line; n = 45).

**Figure 2:**
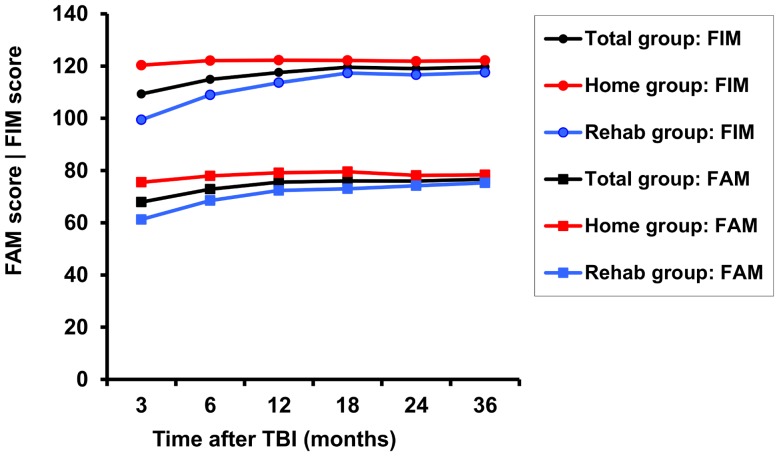
Estimated mean Functional Independence Measure (FIM) and Functional Assessment Measure (FAM) scores over time in the Rehab group (blue line; n = 53), Home group (red line; n = 45), and total group (black line; n = 98).

At 3 years post-TBI, the number of responders for WSRS, FIM and FAM scores was 88 (90%), 95 (97%), and 94 (96%), respectively, due to loss to follow-up or missing data. Figure 1 shows the course of mood and Figure 2 the course of FIM and FAM scores over time in the Home, Rehab and total group. Mean WSRS scores of patients who were initially discharged from the hospital to a rehabilitation center (n = 41) or a nursing home (n = 12) did not differ significantly (p = 0.684) and were therefore united into one group, the Rehab group.

The mean mood score did not change until 18 months after TBI, after which it showed a significant improvement in both groups (compared to the scores at 18 months, at 24 months there was a 24% decline in WSRS score in the Rehab group and 30% in the Home group). The mean FIM and FAM scores improved up to respectively 18 and 12 months after TBI and remained stable thereafter.

Table 2 shows the mean WSRS, FIM and FAM scores and the proportion of normal, borderline and patients with a mood disturbance in both groups and in the total group from 3 months post-injury to 36 months post-injury. The mean WSRS scores 3 years after TBI were in the normal range in 86% of the patients, 5% had borderline scores and 9% had a mood disturbance.

**Table 2:** FIM, FAM and WSRS outcomes at 3, 6, 12, 18, 24 and 36 months after TBI.

		3 months	6 months	12 months	18 months	24 months	36 months
**FIM (mean)**	*Rehab*	99	109	114	117	117	118
	*Home*	120	122	122	122	122	122
	*Total*	109	115	118	120	119	120
**FAM (mean)**	*Rehab*	61	69	72	73	74	75
	*Home*	76	78	79	80	78	78
	*Total*	68	73	76	76	76	77
**WSRS (mean)**	*Rehab*	5.9	6.0	6.0	6.4	4.8	4.7
	*Home*	4.2	4.2	3.7	3.6	2.5	2.7
	*Total*	5.0	5.1	5.0	5.0	3.7	3.8
**Normal (%)**	*Rehab*	73	74	74	72	79	79
	*Home*	84	83	90	90	92	95
	*Total*	81	79	82	81	86	86
**Borderline (%)**	*Rehab*	9	9	11	5	11	9
	*Home*	8	8	3	3	3	0
	*Total*	8	8	6	4	7	5
**Mood dist.(%)**	*Rehab*	18	17	16	23	11	13
	*Home*	8	10	8	8	5	5
	*Total*	10	13	12	16	8	9

FIM = Functional Independence Measure; FAM = Functional Assessment Measure;

WSRS = Wimbledon Self-Report Scale; Normal = WSRS score<8; Borderline = WSRS score 8–10; Mood disturbance = WSRS score>10;

Total group N = 98; Rehab group n = 53; Home group, n = 45.

**Figure 3:**
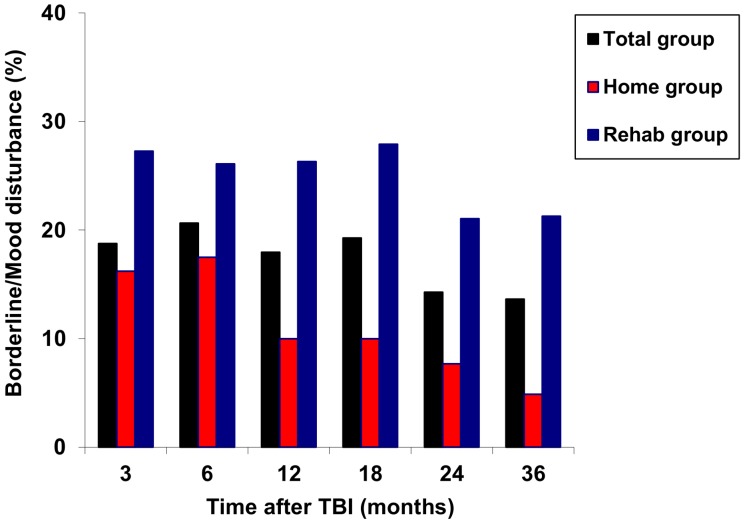
Proportion of patients with a mood disturbance in the Rehab group (blue; n = 53), Home group (red; n = 45) and total group (black; n = 98) over time.

Figure 3 shows the proportions of patients with a borderline or mood disturbance over time in each group.

### Predictors of Mood Disturbance

None of the sociodemographic characteristics nor TBI severity (measured with the GCS) were significant predictors of the mood scores (Table 3). Significant predictors of the mood score, assessed in the home environment, in relation to time (months) after TBI were initial hospital discharge destination, and concurrent FIM and FAM scores. In the multivariable mixed model the FIM score was no longer significant (p = 0.170). The strongest predictors of mood were time after TBI (*β = *−0.03, p = 0.072), FAM score (*β = *−0.28, p<0.001), and discharge destination (*β = *1.27; p = 0.071), the latter indicating that patients who were directly discharged home tended to have better mood scores up to 3 years after TBI, than patients that were initially treated in an inpatient rehabilitation center or nursing home after hospital discharge.

**Table 3:** Results of the mixed model analysis to predict the course of mood measured with the Wimbledon Self-Report Scale (WSRS) total scores in the home environment.

Predictor	β	SE	p-value
Time, months after TBI	−0.04	0.02	0.008#
**Sociodemographic characteristics**			
Age, years	0.05	0.03	0.108
Gender			
*Male*	0.24	0.92	0.796
*Female* *	0	0	
Marital status			
*Single*	−0.39	0.58	0.502
*Married/living together* *	0	0	
Work status			
*Unemployed*	0.09	0.43	0.826
*Employed* *	0	0	
Educational level			
*Low*	0.07	0.70	0.919
*High*	0	0	
**Clinical characteristics**			
Severity TBI			
*Moderate (GCS 9–13)*	0.20	0.97	0.836
*Severe (GCS 3–8)* *	0	0	
Length of stay in hospital, days	0.02	0.02	0.132
**Post-acute functioning**			
Hospital discharge destination:			
*Inpatient setting (Rehab group)*	2.13	0.81	0.010#
*Home* *	0	0	
FIM	−0.22	0.04	<0.001
FAM	−0.29	0.04	<0.001#

Results are presented as univariable regression coefficients (β) with standard errors (SE) and p-values.

*This parameter is set as the reference category.

# Predictors included in the final multivariable regression model (p<0.10).

SE: standard error; TBI: traumatic brain injury; GCS: Glasgow Coma Scale; FIM: Functional Independence Measure; FAM: Functional Assessment Measure.

### Comparison between Responders and Non-responders

To investigate possible differences between the responders and patients that were not measured or lost to follow-up, we compared the age at injury, GCS score, and LOS of these two groups at 3 and 36 months follow-up (Table 4).

**Table 4:** Comparison between characteristics (mean (SD)) of patients measured and of patients not measured at 3 and 36 months after TBI.

	Patients measured	Patients not measured	p-value
**WSRS at 3 months, n**	48	50	
Age at injury, years	30.3 (12.2)	35.1 (13.3)	0.066
GCS	7.71 (2.70)	5.51 (2.11)	<0.001
LOS, days	24 (12)	54 (31.8)	<0.001
**WSRS at 36 months, n**	88	10	
Age at injury, years	32.7 (13.1)	33.2 (11.7)	0.913
GCS	6.53 (2.66)	7.30 (2.41)	0.386
LOS (days)	39 (27)	46 (39.8)	0.421
**FIM at 3 months, n**	89	9	
Age at injury, years	32.4 (13.1)	36.8 (10.0)	0.331
GCS	6.62 (2.67)	6.56 (2.40)	0.946
LOS (days)	37 (24)	64 (54)	0.183
**FIM at 36 months, n**	91	7	
Age at injury, years	33.0 (13.1)	30.0 (10.6)	0.557
GCS	6.53	7.71 (1.50)	0.253
LOS (days)	41 (29)	26 (20)	0.188

GCS = Glasgow Coma Scale, LOS = Length of hospital stay, FIM = Functional Independence Measure, WSRS = Wimbledon Self-Report Scale.

WSRS scores were only measured if patients were finally discharged to their homes, thus most of the non-responders are found at the first follow-up measurement. Three months post-TBI, patients with WSRS scores were significantly younger, had a better GCS score and a shorter LOS in hospital compared to the patients whose WSRS was not measured. After 36 months the differences in baseline values between patients with and without WSRS scores were no longer significant.

At 3 and 36 months post-TBI there were no significant differences between patients with FIM scores and patients with missing FIM scores.

## Discussion

Using a prospective design, the present study evaluates the long-term course of mood after moderate or severe TBI. Mood scores as measured with the WSRS in the home environment were stable in the first 18 months post-onset, improved thereafter and stabilized after 2 years. Because only patients with moderate and severe TBI were included, we expected a higher prevalence of mood disorders. In the first 12 to 18 months patients showed considerable improvement on motor and cognitive functioning as measured with the FIM and FAM scores respectively; after this period of time the FIM and FAM scores stabilized. Time after TBI, initial hospital discharge destination and FAM scores were identified as the strongest predictors of mood. None of the sociodemographic characteristics nor TBI severity (as measured with the GCS) predicted mood disturbances on the long term. This is consistent with findings from others [6], [7], [12], [22].

A mood disturbance rate of 9% at 3 years after moderate or severe TBI is low in relation to other reports. Even if borderline symptoms were included as (mild) mood disturbance (total mood disturbance rate 14%) or if we only look at the rehabilitation group (mood disturbance rate 21%) our numbers are lower than those reported earlier. For example, Hoofien et al. established high rates of depression (45.3%) at (on average) 14.1 years after severe TBI [10]. Andelic et al. found a depression rate of 31% 10 years after moderate and severe TBI [23]. However, these studies suffered from a retrospective design in which selection bias may have played a role. When comparing our results with data from the prospective study of Dikmen et al., in which 17% of the moderate to severe TBI patients was depressed after 3–5 years [11], our rate is similar. Furthermore, in our study patient inclusion took place in the hospital and was not restricted to the rehabilitation center, as is the case in most studies. Therefore, our data may better represent mood disturbance rates in the total moderate to severe TBI population. It should be noted, however, that in the present study mood was only measured if the patient was staying at home at the time of measurement; thus patients still residing in an inpatient setting were excluded so that institutional factors would not influence the mood scores. On the long term, mood scores of patients initially discharged to inpatient rehabilitation remained worse than mood scores from patients directly discharged home, taking into account functional status. For comparison, functional status at 3 months was assessed in all patients, institutionalized or not. For functional status we found no significant differences between responders and non-responders at any point in time. Therefore, our results may imply that it might be favourable to promote a fast discharge home to prevent mood disorders. However, early discharge home places a greater demand on outpatient rehabilitation services and on caregivers’ support [24]. In addition, mood disorders during hospitalization might co-determine discharge destination. Unfortunately, specific data on psychological or pharmacological treatment of mood disturbance were not collected during the course of this study. We can therefore not exclude the influence caused by treatment of mood disturbances on the outcomes. Even if patients with mood disturbances during hospitalization are more likely to being discharged to a rehabilitation center or nursing home, these patients still tend to have worse mood scores up to 3 years after TBI. Therefore, it is important to monitor mood on a regularly basis in patients with moderate to severe TBI, especially in these settings, and to treat symptoms of depression if necessary.

In the present study the FIM scores improved up to 18 months post-TBI and the FAM scores up to 12 months post-TBI after which scores stabilized; the mood scores started to improve after 18 months and stabilized after 2 years. Thus, mood seems to improve when recovery in the motor and cognitive domain have stabilized.

Schönberger et al. also studied the relation between depression and functional outcome using the Structured Clinical Interview for DSM-IV (SCID) and the Extended Glasgow Coma Scale (GOS-E) measured at 6 and 12 months post-TBI. The occurrence of depression between 6 and 12 months post-injury was predicted by the functional status 6 months post-injury [25].

Significant correlations between the Hospital Anxiety and Depression Scale (HADS) and the Community Outcome Scale (COS) and between COS and items of the FIM/FAM have also been demonstrated; however, no information was available on the concurrent relationship between depression and functional status [26]. Ponsford et al. investigated the relationship between psychiatric state and functional outcome at 10 years after TBI using the HADS and the GOS-E; in that retrospective study the relation between greater depression and poorer outcome on the GOS-E only approached significance [27]. The present study with a prospective design and a relatively long follow-up period indicates that functional status (FIM and FAM scores), especially cognitive functioning (FAM score), remains associated with mood up to 3 years post-injury.

### Conclusions

Mood starts to improve 18 months after TBI when motor and cognitive outcome have stabilized. Time post TBI, cognitive outcome and initial discharge destination are the strongest predictors of mood up to 3 years after TBI. Therefore, mood scores of patients with moderate to severe TBI should be frequently monitored, especially in rehabilitation centers and nursing homes. For future TBI research we recommend a prospective multicenter study, measuring mood scores in both inpatients and outpatients over time, to confirm our results.

## References

[1] Andlin-Sobocki P, Jonsson B, Wittchen HU, Olesen J (2005) Cost of disorder of the brain in Europe. Eur J of Neurol 12: 1–27. 10.1111/j.1468-1331.2005.01202.x 15877774

[2] Tagliaferri R, Compagnone C, Korsic M, Servadei F, Kraus J (2006) A systematic review of brain injury epidemiology in Europe. Acta Neurochir (Wien) 148: 255–268. 16311842 10.1007/s00701-005-0651-y

[3] Thurman DJ, Alverson C, Dunn KA, Guerrero J, Sniezek JE (1999) Traumatic brain injury in the United States: a public health perspective. J Head Trauma Rehabil 14: 602–615. 10671706 10.1097/00001199-199912000-00009

[4] Kennedy RE, Livingston L, Riddick A, Marwitz JH, Kreutzer JS, et al (2005) Evaluation of the Neurobehavioural Functioning Inventory as a depression screening tool after traumatic brain injury. J Head Trauma Rehabil 20: 512–526. 16304488 10.1097/00001199-200511000-00004

[5] Rosenthal M, Christensen BK, Ross TP (1998) Depression following traumatic brain injury. Arch Phys Med Rehabil 79: 90–103. 9440425 10.1016/s0003-9993(98)90215-5

[6] Bombardier CH, Fann JR, Temkin NR, Esselamn PC, Barber J, et al (2010) Rates of major depressive disorder and clinical outcomes following traumatic brain injury. JAMA 303: 1938–1945. 20483970 10.1001/jama.2010.599 PMC3090293

[7] Hart T, Brenner L, Clark AN, Bogner JA, Novack TA, et al (2011) Major and minor depression after traumatic brain injury. Arch Phys Med Rehabil 92: 1211–1219. 21807140 10.1016/j.apmr.2011.03.005

[8] Bowen A, Neumann V, Conner M, Tennant A, Chamberlain MA (1998) Mood disorders following traumatic brain injury: identifying the extent of the problem and the people at risk. Brain Inj 12: 177–190. 9547948 10.1080/026990598122656

[9] Kim E, Lauterbach EC, Reeve A, Arciniegas DB, Coburn KL, et al (2007) Neuropsychiatric complications of traumatic brain injury; a critical review of the literature (a report by the ANPA committee on research). J Neuropsychiatry Clin Neurosci 19: 106–127. 17431056 10.1176/jnp.2007.19.2.106

[10] Hoofien D, Gilboa A, Vakil E, Donovic PJ (2001) Traumatic brain injury (TBI) 10–20 years later: a comprehensive outcome study of psychiatric symptomatology, cognitive abilities and psychological functioning. Brain Inj 15: 189–209. 11260769 10.1080/026990501300005659

[11] Dikmen SS, Bombardier CH, Machamer JE, Fann JR, Temkin NR (2004) Natural history of depression in traumatic brain injury. Arch Phys Med Rehabil 85: 1457–1464. 15375816 10.1016/j.apmr.2003.12.041

[12] Jorge RE, Starkstein SE (2005) Pathophysiologic aspects of major depression following traumatic brain injury. J Head Trauma Rehabil 20: 475–487. 16304485 10.1097/00001199-200511000-00001

[13] van Baalen B, Odding E, van Woensel MPC, van Kessel MA, Roebroeck ME, et al (2006) Reliability and sensitivity to change of measurement instruments used in a traumatic brain injury population. Clin Rehabil 20: 686–700. 16944826 10.1191/0269215506cre982oa

[14] Willemse-van Son AH, Ribbers GM, Hop WC, Stam HJ (2009) Community integration following moderate to severe traumatic brain injury: a longitudinal investigation. J Rehabil Med 41: 521–527. 19543662 10.2340/16501977-0377

[15] Teasdale G, Jennett B (1974) Assessment of coma and impaired consciousness. Lancet 13: 81–84. 10.1016/s0140-6736(74)91639-0 4136544

[16] Coughlan AK, Storey P (1988) The Wimbledon SelfReport Scale: emotional and mood appraisal. Clin Rehabil 2: 207–213.

[17] van Balen HG (1992) Mensen met een traumatisch hersenletsel: probleeminventarisatie. ‘Persons with traumatic brain injury: problem assessment.’ Department of Research and Development, Radboud University Nijmegen, the Netherlands.

[18] Stineman MG, Shea JA, Jette A, Tassoni CJ, Ottenbacher KJ, et al (1996) The functional independence measure: tests of scaling assumptions, structure, and reliability across 20 diverse impairment categories. Arch Phys Med Rehabil 77: 1101–1108. 8931518 10.1016/s0003-9993(96)90130-6

[19] MacPherson KM, Pentland B, Cudmore SF, Prescott RJ (1996) An interrater reliability study of the Functional Assessment Measure (FIM+FAM). Disabil Rehabil 18: 341–347. 8799674 10.3109/09638289609165892

[20] Hawley CA, Taylor R, Hellawell DJ, Pentland B (1999) Use of the functional assessment measure (FIM+FAM) in head injury rehabilitation: a psychometric analysis. J Neurol Neurosurg Psychiatry 67: 749–754. 10567491 10.1136/jnnp.67.6.749 PMC1736663

[21] Donaghy S, Wass PJ (1998) Interrater reliability of the Functional Assessment Measure in a brain injury rehabilitation program. Arch Phys Med Rehabil 79: 1231–1236. 9779676 10.1016/s0003-9993(98)90267-2

[22] Malec JF, Brown AW, Moessner AM, Stump TE, Monahan P (2010) A preliminary model for posttraumatic brain injury depression. Arch Phys Med Rehabil 91: 1087–1097. 20599048 10.1016/j.apmr.2010.04.002

[23] Andelic N, Hammergren N, Bautz-Holter E, Sveen U, Brunborg C, et al (2009) Functional outcome and health-related quality of life 10 years after moderate-to-severe traumatic brain injury. Acta Neurol Scand 120: 16–23. 18976326 10.1111/j.1600-0404.2008.01116.x

[24] Hawkins ML, Lewis FD, Medeiros RS (2005) Impact of length of stay on functional outcomes of TBI patients. Am Surg 71: 920–929. 16372610

[25] Schönberger M, Ponsford J, Gould KR, Johnston L (2001) The temporal relationship between depression, anxiety, and functional status after traumatic brain injury; a cross-lagged analysis. J Int Neuropsychol Soc 17: 781–787. 10.1017/S1355617711000701 21729404

[26] Stilwell P, Stilwell J, Hawley C, Davies C (1998) Measuring outcome in community-based rehabilitation services for people who have suffered traumatic brain injury: the community outcome scale. Clin Rehabil 12: 521–531. 9869256 10.1191/026921598673761855

[27] Ponsford J, Draper K, Schönberger M (2008) Functional outcome 10 years after traumatic brain injury: its relationship with demographic, injury severity, and cognitive and emotional status. J Int Neuropsychol Soc 14: 233–242. 18282321 10.1017/S1355617708080272

